# Mid- and Longterm Neo-Aortic Valve Regurgitation after Jatene Surgery: Prevalence and Risk Factors

**DOI:** 10.5935/abc.20180111

**Published:** 2018-07

**Authors:** Cristiane Nunes Martins, Bayard Gontijo Filho, Roberto Max Lopes, Francisco das Chagas Lima e Silva

**Affiliations:** 1 BIOCOR Hospital de Doenças Cardiovasculares, Belo Horizonte, MG - Brazil; 2 Hospital Santa Casa de Belo Horizonte, Belo Horizonte, MG - Brazil

**Keywords:** Heart Defects, Congenital, Transposition of Great Vessels, Transposition of Large Vessels, Aortic Valve Insufficiency

## Abstract

**Background:**

Jatene surgery became the surgical procedure of choice to repair transposition of the great arteries (TGA) in neonates and infants. Late complications, mainly related to the pulmonary outflow tract and coronary arteries, are well known. The behavior of the neo-aortic valve is a cause of concern because of its potential for requiring late reoperation.

**Objectives:**

To assess the prevalence and risk factors of neo-aortic valve regurgitation in 127 patients in the late postoperative period of the Jatene surgery.

**Methods:**

Of the 328 survivors of the Jatene surgery at the Biocor Institute from October 1997 to June 2015, all patients undergoing postoperative follow-up were contacted via telephone, 127 being eligible for the study. The patients were divided into two groups, simple TGA and complex TGA groups, with follow-up means of 6.4 ± 4.7 years and 9.26 ± 4.22 years, respectively. Echocardiography was performed with adjusted measurements (Z-score) of the neo-aortic annulus, sinus of Valsalva, sinotubular region and ascending aorta, as well as quantification of the neo-aortic valve regurgitation grade.

**Results:**

The incidence of mild neo-aortic valve regurgitation was 29% in a follow-up of 7.4 ± 4.7 years. Moderate regurgitation was identified in 24 patients with age mean (± standard-deviation) of 9.81 ± 4.21 years, 19 of whom (79%) in the complex TGA group. Those patients had a higher aortic annulus Z-score. The reoperation rate due to neo-aortic regurgitation associated with aortic dilation was 1.5%, all patients in the complex TGA group.

**Conclusion:**

This study shows that, despite the low incidence of reoperation after Jatene surgery due to neo-aorta dilation and neo-aortic valve regurgitation, that is a time-dependent phenomenon, which requires strict vigilance of the patients. In this study, one of the major risk factors for neo-aortic valve regurgitation was the preoperative pulmonary artery diameter (p < 0.001).

## Introduction

Transposition of the great arteries (TGA) has been known for almost 300 years.^1^ In 1797, Matthew Baille described a condition in which the aorta originated from the right ventricle and the pulmonary artery, from the left ventricle.^2^ In 1814, Farré used the term “transposition” to characterize the malformation described by Baillie. The history of the surgical correction of TGA begins in the 1950s with palliative procedures, progressing to techniques of atrial correction (Mustard/Senning).^3^

The surgical treatment of TGA was modified with the publication of the anatomical correction technique by Adib Jatene^4^ in 1976, changing patients’ outcome. Throughout the years, thus, the Jatene surgery has been established as the arterial switch operation of choice, with complete physiological and anatomical corrections. Its superiority has been corroborated by long-term results showing the preservation of good left ventricular (LV) function^5^ and sinus rhythm, as well as low mortality, with a survival rate over 88% in the 10-to-15-year follow-up.^6^

Complications are not frequent in the immediate postoperative period, being mainly related to the patient’s preoperative condition, prolonged cardiopulmonary bypass duration and coronary artery obstruction, with consequent myocardial ischemia. Despite the excellent clinical outcome of most patients in the mid and long run,^5^ the rate of late reoperation is significant after the Jatene surgery. The major reasons for reintervention are right ventricular (RV) outflow tract and coronary obstructions and progressive neo-aorta dilation associated with aortic regurgitation. Although technical modifications have determined a significant reduction in reinterventions for RV outflow tract^7^ and coronary obstructions,^8^ the late progression of neo-aorta dilation and neo-aortic valve regurgitation is of great concern.

This study was aimed at investigating the factors that could contribute to the progression of neo-aortic valve regurgitation by use of a retrospective review of a group of patients who had had surgery at a single institution.

## Methods

From October 1997 to June 2015, 367 patients with TGA were submitted to the Jatene surgery at the Biocor Institute of Cardiovascular Diseases from Minas Gerais, 328 of whom survived and were discharged from the hospital. This observational study was performed from November 2015 to May 2016 at the Biocor Institute as part of a Master’s thesis. Of the 328 survivors, 251 were on regular outpatient follow-up, and 127 participated in this study, being divided into two groups based on their anatomical characteristics.

In the simple TGA group, 84 patients with TGA and intact ventricular septum were included.

The complex TGA group included 43 patients with TGA and intermediate to large ventricular septal defect (VSD) and patients with double RV outflow tract without pulmonary stenosis (Taussig Bing), with or without obstruction of the aortic arch.

Patients with the following characteristics were excluded from the study: children with a postoperative period shorter than 2 years (n = 18); patients submitted to ventricular preparation (n = 3); patients submitted to pulmonary artery reduction plasty (n = 27), a technique for patients with great disproportion in the sizes of the neo-aorta and neo-pulmonary artery, which began to be used at the Biocor Institute in 2006; and those who could not attend the consultations (n = 76). Seventy-five patients were lost to follow-up and two had late death.

### Preoperative data collection

The medical records were reviewed for collection of pre-, perioperative and immediate postoperative demographic data, such as anatomical characteristics of the defect, age in days and body surface at the time of surgical correction, adjusted pulmonary artery measurement, and presence of associated anomalies.

### Postoperative data collection

During postoperative assessment, all patients underwent clinical examination by a pediatric cardiologist of the institution, with weight and height measurement to calculate body surface. Transthoracic echocardiography was performed with no cost to the patient. The Secretariats of Health of the respective municipalities were responsible for the patients’ transportation, and when that was not available, this study’s author responded to that need. This study was approved by the local Ethics Committee, in accordance with the Declaration of Helsinki, regarding research in human beings. All individuals or their legal guardians provided written consent for this study.

### Surgical technique

The Jatene surgery technique used at the Biocor Institute was the same during the entire study period. Lecompte maneuver was used for almost all patients (96%) and coronary reimplantation was performed with the neo-aorta distended and always in the sinuses of Valsalva, never in the suture line (“trap door”). The approach to the VSD varied according to its anatomical location: via the right atrium, aorta or pulmonary artery. Pulmonary reconstruction was performed with autologous pericardium (two patches or monopatch).

### Methodology of the echocardiographic study

The echocardiographic study was performed by the author, the pediatric echocardiographer at the Biocor Institute, with a Phillips HD11 device and four sequential measurements of the aorta, quantifying the neo-aortic valve regurgitation grade. Another equally trained echocardiographer performed the same exam, and the measurements were compared.

There was no discrepancy between the echocardiographers regarding the measurements. Thus, no other checking was necessary, because the guidelines regarding measurements are very clear.^9^

**Figure 1:**
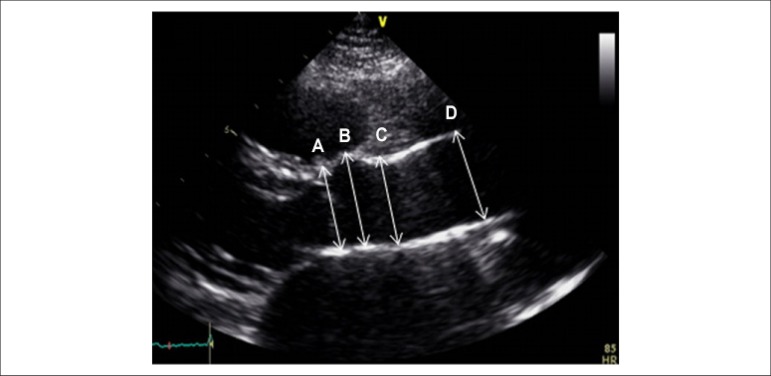
Measurements of the aorta. Two-dimensional echocardiogram of the neo-aorta, parasternal view of the long axis of the left ventricle. The measurement sites are shown: A- aortic annulus; B- sinus of Valsalva; C- sinotubular region; D- ascending aorta.

Serial measurements of the neo-aortic annulus, sinus of Valsalva, sinotubular region and ascending aorta were taken in the parasternal view of the long axis of the left ventricle and adjusted for body surface, following the American Society of Echocardiography (ASE) guidelines (Figure 1). In accordance with those guidelines, the aortic root was considered to extend from the implantation of the aortic leaflets in the LV outflow tract to the tubular portion of the aorta (sinotubular junction).^9^

The aortic root is a geometrically complex structure that includes: (1) aortic valve annulus; (2) interleaflet triangles; (3) semilunar aortic leaflets and their attachments; (4) sinuses of Valsalva; (5) sinotubular junction.^10^

The aortic measurements were taken at the following sites: (1) aortic valve annulus; (2) maximum diameter of the sinus of Valsalva; (3) sinotubular junction (usually a well-defined transition between the sinuses of Valsalva and the tubular portion of the ascending aorta); (4) maximum diameter of the proximal ascending aorta, recording the distance between the measurement site and the sinotubular junction.^9^

The measurements of the aortic annulus, sinus of Valsalva, sinotubular region and ascending aorta were adjusted by using the Z-score.^11,12^ Similarly, the measurements of the aortic annulus were taken in accordance with the ASE recommendations.^9^ Thus, they were taken in the zoom mode, in mid systole, when the annulus is slightly larger and rounder than in diastole, between the hinging points of the aortic valve leaflets (usually between the hinging point of the right coronary leaflet and the border of the sinus at the side of the commissures between the left coronary leaflet and the noncoronary leaflet) in its internal border. In accordance with the ASE recommendations, all other aortic measurements were taken at the end of diastole, along a strictly perpendicular plane to the long axis of the aorta.^9^

The neo-aortic valve regurgitation was assessed on color Doppler echocardiography and quantified as absent or trivial, mild, moderate and severe, depending on the relationship between the regurgitating jet and the LV outflow tract diameter.^13^ If that relationship was smaller than 0.25, the regurgitation was quantified as mild; if between 0.25 and 0.5, moderate; and if higher than 0.5, severe. However, considering the possibility of underestimating the regurgitation grade in patients with aortic annulus dilation, the flow in the descending aorta was analyzed by use of Doppler echocardiography. In the presence of holodiastolic flow reversal in the descending aorta, regurgitation was considered moderate or severe.^14^

The regurgitation grade was compared to the neo-aorta diameter in its respective measurements.

The body surface was calculated by using the Mosteller formula: A = √ (height x weight) / 3600).^15^

### Statistical analysis

The Epi Info software, version 6, was used for data collection and database management. The Epi Info and Microsoft Office Excel, version 2000 were used for the statistical analyses, the latter being also used to elaborate and edit the tables.

The categorical variables were compared by using Pearson chi-square and Fisher Exact tests, when necessary. To compare the means of continuous variables, Student *t* test was used for independent samples when the distribution was normal, paired *t* test was used for paired samples, and Kruskal-Wallis test was used to compare the medians.

Analysis of variance (ANOVA) was used to compare several groups of continuous variables at one time.

A p value < 0.05 was adopted for statistical significance.

## Results

### Analysis of the preoperative characteristics of the simple and complex TGA groups

Table 1 shows the following pre- and perioperative characteristics of the two groups: sex, body surface, age and pulmonary annulus adjusted to body surface.

**Table 1:** Means, standard deviations and medians of the patients of the simple TGA and complex TGA groups submitted to Jatene surgery

Variables	n	Mean ± standard-deviation	Median	Statistical test
**Sex**				
**Simple TGA**				
Male	60			Chi-square = 0.83
Female	24			
**Complex TGA**				
Male	30			
Female	13			
**Body surface**				
Simple TGA	84	0.20 ± 0.04 m^2^	0.20	p = 0.86
Complex TGA	43	0.21 ± 0.08 m^2^	0.20	
**Pulmonary annulus Z-score**				
Simple TGA	84	1.6 ± 0.6	0.2 ± 0.3	p = 0.18
Complex TGA	43	1.9 ±1.1	0.3 ± 0.4	

(*) Statistically significant difference.

Of the 127 patients assessed, 84 were in the simple TGA group and 43, in the complex TGA group. The follow-up duration was 7.4 ± 4.7 years.

The body surface means were 0.20 ± 0.04 m^2^ and 0.21 ± 0.08 m^2^ for the simple TGA and complex TGA groups, respectively.

When comparing the preoperative Z-score of the pulmonary annulus (Table 1), the complex TGA group had the highest Z-score, a finding of statistical significance.

Table 2 shows the associated anomalies found in 21 patients (16.5%), the most frequently one being the aortic arch anomaly, identified in 7 patients (5.5%), 6 of which in the complex TGA group.

**Table 2:** Associated anomalies in the simple TGA and complex TGA groups

Associated anomalies (n = 21)	Simple TGA	Complex TGA
Dextrocardia *situs solitus*	2	0
Dextrocardia *situs inversus*	0	1
Juxtaposition of atrial appendages	3	1
L-position of the aorta	0	3
Tricuspid straddling	0	2
Superoinferior ventricle	0	2
Aortic arch anomalies	1	6
TOTAL	6	15

### Analysis of the postoperative characteristics of the simple TGA and complex TGA groups

**Analysis 2.1 -** Table 3 shows that the mean ages for the simple TGA group and the TGA with VSD group were 6.4 ± 4.73 years and 9.26 ± 4.22 years, respectively.

**Table 3:** Means and standard deviations of the ages of the patients of the simple TGA and complex TGA groups at the time of control

Variables	n	Mean ± standard deviation	Statistical test	P value
**Age in control**				
Simple TGA	84	6.40 ± 4.73 anos	Student *t* test	< 0.0001
Complex TGA	43	9.26 ± 4.22 anos		(*)

*t = 3.34

**Analysis 2.2 -** Table 4 compares the preoperative Z-score mean of the pulmonary artery with the postoperative Z-score mean of the neo-aorta of 84 patients in the simple TGA group and 43 patients in the complex TGA group, showing a statistically significant difference between the means.

**Table 4:** Comparison between the preoperative Z-score mean of pulmonary artery and the postoperative Z-score mean of aortic annulus of the simple TGA and complex TGA groups

Z-score (Complex TGA)	Mean ± standard-deviation	Difference between the means	Paired *t* test	P value
Pre	1.9 ± 1.1	-0.3 ± 0.4	4.88	< 0.0001
Post	2.2 ± 1.3			
**Z-score (Simple TGA)**	**Mean ± standard-deviation**	**Difference between the means**	**Paired *t* test**	**P value**
Pre	1.6 ± 0.7	-0.2 ± 0.3	4.81	< 0.0001
Post	1.8 ± 1.0			

*p < 0.0001

**Analysis 2.3 -** Table 5 shows no or trivial neo-aortic valve regurgitation in 74 patients of the simple TGA group (88%) and in 16 patients of the complex TGA group (37.2%). Mild regurgitation was observed in 5 patients of the simple TGA group (5.9%) and in 8 patients of the complex TGA group (18.6%). Moderate regurgitation was identified in 5 patients of the simple TGA group (5.9%) and in 19 patients of the TGA with VSD group (44.8%). Absent or trivial regurgitation predominated in the simple TGA group (p < 0.0001).

**Table 5:** Assessment of the neo-aortic valve regurgitation grade of 127 patients of the simple TGA and complex TGA groups submitted to Jatene surgery

Groups	Absent/trivial regurgitation	Mild regurgitation	Moderate regurgitation	Total
Simple TGA	74 (82.2%)	5 (38.4%)	5 (20.8%)	84
Complex TGA	16 (17.7%)	8 (61.5%)	19 (79.1%)	43
Total	90 (70.8%)	13 (10.2%)	24 (18.9%)	127

Chi-square = 34.85; p < 0.0001

**Analysis 2.4 -** In patients with no or trivial regurgitation (90 patients), the aortic annulus Z-score mean ± standard-deviation was 1.72 ± 0.98 cm. In patients with mild regurgitation (13 patients), the Z-score mean ± standard-deviation was 2.18 ± 0.83 cm, and, in those with moderate regurgitation (24 patients), 2.60 ± 1.40 cm. The results show that, the larger the aortic annulus, the higher the regurgitation grade (p < 0.001).

**Analysis 2.5 -** Table 7 shows that patients submitted to the Jatene surgery with a moderate neo-aortic valve regurgitation had a higher age mean (p=0.0145). Of the 127 patients studied, 2 patients in the complex TGA group required valve change because of regurgitation progression during data collection, the reoperation rate being 1.5%.

**Table 7:** Age means at the control times according to the neo-aortic valve regurgitation grade during the follow-up of 127 patients submitted to Jatene surgery

Regurgitation grade	n	Mean ± standard-deviation	Analysis of variance	F
Absent/trivial	90	7.08 ± 4.74 anos	F	5.4
Mild	13	5.60 ± 4.16 anos		
Moderate	24	9.81 ± 4.21 anos		

F = 5.4

## Discussion

Reintervention due to neo-aortic valve regurgitation after Jatene surgery was first reported in 2009 in a 16-year-old adolescent.^16^ That report concluded that neo-aorta dilation was present in two thirds of the patients and that moderate regurgitation was observed in 15%, emphasizing the need for careful follow-up in that group of patients.

Although the overall incidence of the surgery for neo-aorta dilation and neo-aortic valve regurgitation after Jatene surgery in 10 years is still low (2-2.5%),^6,16-19^ several authors have reported that the development of neo-aorta regurgitation and dilation is a time-dependent phenomenon, requiring a strict vigilance of the patients.^6,20,21^ McMahon et al.^22^ have found a moderate enlargement in the neo-aortic root (Z-score between 3 and 4) in 52% of the patients, and a severe enlargement of the neo-aortic root (Z-score > 5) in 25%. In addition, those authors have shown that the development of significant neo-aortic valve regurgitation strongly associated with the development of neo-aorta dilation, which has been confirmed by other authors.^23^ Schwartz et al.^24^ have concluded that, after Jatene surgery, neo-aortic root dilation and neo-aortic valve regurgitation continue to develop over time, but aortic root dilation does not tend to be progressive during late follow-up. However, in that series, the last follow-up was up to 16 years, while Walter et al.^19^ have concluded that neo-aortic regurgitation can develop in up to 15 years. In our sample, two patients required reintervention for progressive neo-aortic root dilation associated with neo-aortic regurgitation in a follow-up of 9.81 ± 4.21 years. Some studies have shown the significance of several risk factors to the development of late neo-aortic valve regurgitation and aortic root dilation, such as preoperative pulmonary artery dilation, patient’s age over one year at the time Jatene surgery is performed, and presence of VSD and complex TGA,^6,18-21^ but such findings could not be repeated in some other large series.^8,23^ In our series, as in others,^6,21,23^ we observed that the most relevant factor for neo-aortic regurgitation was the great disproportion in the sizes of the neo-aorta and neo-pulmonary artery at the time of surgery, which was present in the complex TGA group, especially when aortic arch anomalies were associated. 

In addition, the VSD found in the complex TGA group is related to two factors that increase the risk for developing valvular regurgitation, pulmonary root dilation and pulmonary artery pressure elevation, which can change the arrangement of the muscle fibers and generate permanent disarrangement of the pulmonary artery, even after anatomical correction.^25^

The presence of neo-aortic valve regurgitation in patients without risk factors, such as simple TGA, can be explained in histopathological studies revealing a reduction in the amount of collagen in the arterial roots in hearts with TGA as compared to that of normal hearts, in addition to less extensive anchorage and embedding of both arterial roots in the myocardium.^26^ The pulmonary root dilation can be compared to that observed after the Norwood surgery for hypoplastic left heart syndrome,^27^ indicating the pulmonary artery included in the systemic circulation is a risk factor per se. From the morphological and histological viewpoints, the pulmonary and aortic valves are indistinguishable at birth. In normal hearts, studies have shown gross and microscopic changes in those valves, probably due to pressure changes resulting from the transition from the fetal to post-natal circulation, resulting in pulmonary valve with thin leaflets, less collagen and a smaller amount of elastic tissue. After surgical repair, the more delicate valve is integrated into the systemic circulation and can be damaged by the high-pressure regime.^27^

Briefly, the etiology of neo-aortic valve regurgitation and neo-aorta dilation is very likely multifactorial. In addition to external risk factors, there are intrinsic structural problems of the pulmonary root integrated into the systemic circulation. Thus, according to our clinical observations, the increase in the number of surgical interventions to treat aortic root dilation and neo-aortic valve regurgitation should be the reason for the constant monitoring of patients with or without additional risk factors.

The present study, similarly to others,^21-23^ showed that the most relevant factor for neo-aortic valve regurgitation was the disproportion in the sizes of the neo-aorta and neo-pulmonary artery at the time of surgery, which was present in the complex TGA group, especially when associated with aortic arch anomalies.

## Conclusion

In the present study, the complex TGA group had a higher preoperative pulmonary artery Z-score as compared to that of the simple TGA group, and a higher incidence of associated anomalies, such as aortic arch anomalies (p = 0.0064). In addition, the neo-aorta dilation is maintained in the postoperative period.

Our results showed that the larger the aortic annulus, the higher the regurgitation grade (p < 0.001). In addition, moderate regurgitation was associated with a higher mean age (p = 0.0145) in both simple TGA and complex TGA groups, indicating the need for the constant monitoring of the patients.

### Limitation

The present retrospective study results from the data collection of two groups of patients with distinct anatomical characteristics, submitted to the same surgical technique.

Some variations related to the presence of aortic regurgitation in the long run reported by other authors (techniques of coronary reimplantation, VSD closure and previous pulmonary artery cerclage) were not approached in this study.

## Figures and Tables

**Table 6:** Z-score means of the aortic annulus according to the regurgitation grade in the postoperative follow-up of 127 patients submitted to Jatene surgery

Regurgitation grade	n	Mean ± standard-deviation	Analysis of variance	F
Absent/trivial	90	1.72 ± 0.98 cm	F	6.66
Mild	13	2.18 ± 0.83 cm		
Moderate	24	2.60 ± 1.40 cm		

F = 6.6
